# A Positive Babinski Reflex Predicts Delayed Neuropsychiatric Sequelae in Chinese Patients with Carbon Monoxide Poisoning

**DOI:** 10.1155/2014/814736

**Published:** 2014-05-15

**Authors:** Jian-Fang Zou, Qiming Guo, Hua Shao, Bin Li, Yuxiu Du, Maofeng Liu, Fengling Liu, Lixin Dai, Min-Hsien Chung, Hung-Jung Lin, How-Ran Guo, Tzu-Meng Yang, Chien-Cheng Huang, Chien-Chin Hsu

**Affiliations:** ^1^Clinical Division of Occupational Medicine, Institute of Occupational Health and Occupational Medicine, Academy of Medical Science, Shandong Province 250000, China; ^2^Division of Toxicology, National Institute of Occupational Health and Poison Control, Beijing 100050, China; ^3^Department of Medicine, Second People's Hospital of Dongying, Shandong Province 250000, China; ^4^Department of Medicine, Qilu Petrochemical Corporation Hospital, Shandong Province 250000, China; ^5^Department of Medicine, Second People's Hospital of Kenli, Shandong Province 250000, China; ^6^Department of Emergency Medicine, Chi-Mei Medical Center, Tainan 710, Taiwan; ^7^Department of Emergency Medicine, Chi-Mei Medical Center, Liouying, Tainan 710, Taiwan; ^8^Department of Biotechnology, Southern Taiwan University of Science and Technology, Tainan 710, Taiwan; ^9^Department of Emergency Medicine, Taipei Medical University, Taipei 110, Taiwan; ^10^Department of Environmental and Occupational Health, Medical College, National Cheng Kung University, Tainan 701, Taiwan; ^11^Department of Occupational and Environmental Medicine, National Cheng Kung University Hospital, Tainan 701, Taiwan; ^12^Department of Child Care and Education, Southern Taiwan University of Science and Technology, Tainan 710, Taiwan

## Abstract

As the human population increased in China, the carbon monoxide is a serious environmental toxin in public health. However, predicting the delayed neuropsychiatric sequelae (DNS) of carbon monoxide poisoning (COP) has not been well studied. We investigated the independent predictors of DNS in patients with COP. This study was conducted at four hospitals in China. Data were retrospectively collected from 258 patients with COP between November 1990 and October 2011. DNS was the primary endpoint. A positive Babinski reflex was the independent predictor for DNS: sensitivity = 53.8% (95% confidence interval [CI]: 26.1–79.6), specificity = 88.6% (95% CI: 83.7–92.1), positive predictive value (PPV) = 20.0% (95% CI: 9.1–37.5), and negative predictive value (NPV) = 97.3% (95% CI: 94.0–98.9). The area under the receiver operating characteristic curve = 0.712 (95% CI: 0.544–0.880). A positive Babinski reflex was very memorable, immediately available, and applicable in clinical practice. Even when the sensitivity and PPV of a positive Babinski reflex were unsatisfactory, it had a good specificity and NPV for excluding the risk of DNS. In patients without a positive Babinski reflex, the risk for DNS was only 2.7%. This finding may help physicians make decisions about dispositions for patients with COP.

## 1. Introduction

As society changes due to the challenge of sustainable development in the face of increased human population especially in the East Asia region, the role of toxicology in enlightened public health and public policy will become even more important. Carbon monoxide poisoning (COP) is common in modern society, resulting in significant morbidity and is one of the leading causes of poisoning death [1]. In the country of East Asia such as Taiwan, COP caused 526 emergency department visits and 55 deaths during 2009–2013 [2]. In People's Republic of China, a 1.35 billion population (59 times than Taiwan and 4.3 times than United States), the number of COP is even harder to estimate. However, because COP is commonly misdiagnosed, the true numbers are likely much higher [3]. Acute COP may induce hypoxic encephalopathy with variable degrees of brain damage, ranging from confusion to deep coma [4]. Approximately one-third of the patients succumb during acute intoxication, and most of the remaining patients recover completely from the first episode [4]. However, 0.2–40% of the survivors develop delayed neuropsychiatric sequelae (DNS) within 2–4 weeks after this pseudo recovery [4, 5]. The main reasons for such a large variation might be different population and variability in choosing patients. The common clinical features of DNS are cognitive changes, sphincter incontinence, akinetic mutism, Parkinsonism, and dystonia [4]. Most patients show prominent improvement in all clinical features, particularly in sphincter incontinence and akinetic mutism [4]. Some sequelae, such as dystonia and cognitive impairments, may persist [4].

The recommended treatment for acute COP is 100 percent normobaric oxygen, commonly delivered from a reservoir through a facemask that prevents rebreathing [6]. Hyperbaric-oxygen therapy is often recommended for patients with acute COP, especially if they have lost consciousness or have severe poisoning [6]. A double-blind randomized study [6] reported that CO-poisoned patients given three hyperbaric oxygen treatments within 24 hours of presentation manifest approximately one-half the rate of neuropsychiatric sequelae at 6 weeks, 6 months, and 12 months after treatment than do those treated with normobaric oxygen. Another study found that hyperbaric oxygen treatment was associated with a significant reduction in the incidence of DNS; however, the success of hyperbaric oxygen treatment may require that it be used within 6 hours after COP [7]. The risk of DNS may also be substantially lowered by prescribing that the patient has at least 12 hours of daily bed rest, not do any stressful physical activity, and not be subjected to stressful medical procedures [8]. However, hyperbaric oxygen has many limitations, such as relative inconvenience, high cost, and the complications of hyperoxic seizures, aural barotrauma, anxiety, and oxidative stress [6]. Identifying patients with a high risk for DNS but who really need hyperbaric oxygen treatment is the most important. In addition, recognizing the independent predictors of a high risk for DNS in patients with COP should help physicians predict the prognosis. Until now, however, the results of predicting the DNS of COP are sparse and inconsistent; thus, prediction seems impractical. One study [9] proposed that elderly patients with more comorbidities, but shorter lucid intervals and fewer dangerous activities of daily living, are more likely to have a poor prognosis. However, “more complications, lucid intervals, and dangerous activities of daily living” are difficult to define. Another study [5] showed that decreased Glasgow Coma Scales level and methemoglobin levels were independent risk factors associated with DNS. However, this study enrolled only pediatric patients, which cannot give us a complete picture of DNS. To clarify this issue, we explored independent mortality predictors in patients with COP in our clinical setting.

## 2. Materials and Methods

### 2.1. Study Design, Setting, Population, and Selection of Participants

Between November 1990 and October 2011, data were retrospectively collected from patients with COP at four hospitals in Shandong Province, People's Republic of China: the Institute of Occupational Health and Occupational Medicine, Second People's Hospital of Dongying, Qilu Petrochemical Corporation Hospital, and Second People's Hospital of Kenli. Patients were enrolled if they had documented exposure to carbon monoxide (elevated carbon monoxide hemoglobin (COHb) level or ambient carbon monoxide concentration) or obvious exposure to carbon monoxide, and if they had any of the following symptoms: loss of consciousness, confusion, headache, malaise, fatigue, forgetfulness, dizziness, visual disturbances, nausea, vomiting, cardiac ischemia, or metabolic acidosis (a calculated base excess lower than −2.0 mmol per liter or a lactate concentration higher than 2.5 mmol per liter). If the COHb level was below 10 percent, the patient was eligible only if COP was the only plausible diagnosis. Patients were excluded if they had a history of neurological disease or psychiatric disorders.

### 2.2. Data Collection and Definition of Variables

All the patients were given 100% oxygen at the time that COP was suspected. The studied hospitals' Human Investigation Committee approved the protocol. The reviewers were blinded to the patients' hospital course and outcomes. Information for a number of variables for each patient was recorded. Any variable not present or equivocal in the patient's medical history or physical exam was considered absent.

Elderly was defined as ≥65 years. We defined an age variable of “>35 years” based on a study [10] which reported that being >35 years old was a risk factor for neuropsychiatric sequelae. Altered mental status was defined as any state of awareness that differs from the normal awareness of a conscious person at admission. Loss of consciousness was defined as a transient loss of consciousness [3]. The lack of a pupil reflex was defined as a lack of response to light stimulation in one eye. A positive Babinski sign was defined as an upturning or extensor plantar response elicited when the sole of the foot was stimulated with a blunt instrument.

The enrolled patients were divided into two groups: (i) without DNS (DNS^−^) and (ii) with DNS (DNS^+^). All the study variables were used for comparisons between groups.

### 2.3. Definition of Endpoint

We used DNS as the primary endpoint. Patients who had a recurrence of original symptoms or developed new symptoms (headache, difficulty concentrating, lethargy, emotional lability, amnestic syndromes, dementia, psychosis, Parkinsonism, chorea, apraxia, agnosia, peripheral neuropathy, urinary incontinence, etc.) [9] after COP were considered DNS^+^ for this analysis.

### 2.4. Data Analysis

All analyses were done using SPSS 16.0 for Windows (SPSS Inc., Chicago, IL, USA). Continuous data are means ±  standard deviation (SD). Comparisons between two groups were made using either an independent-samples* t*-test (assuming normal distribution) or Mann-Whitney-Wilcoxon tests (assuming non-normality) for the continuous variables. Either a *χ* ^2^ test or a Fisher's exact test was used for categorical variables. The significant level was set at *P* < 0.05 (two tailed) in all analyses, but in choosing variables to be included in the multiple logistic regression analysis, a cutoff of 0.1 was applied in order to include more potential covariates. The area under the receiver operating characteristic (ROC) curves was used to compare a predictor's specification.

## 3. Results

The final study cohort consisted of 258 patients (123 men [48%] and 135 women [52%]) after patients who had a neurological or psychiatric history had been excluded (Table 1). Their ages ranged from 6 to 97 years (mean age: 54.9 ± 22.4; median, 59). Thirteen patients (5.03%) had DNS. Two hundred patients (77.5%) had undergone hyperbaric oxygen therapy.

Univariate analysis showed that patients with the following variables had a higher risk for DNS (*P* < 0.05): ischemic stroke history and a positive Babinski reflex (Table 1). Other variables were not significantly different between groups. Multiple logistic regression modeling, using the univariate comparison with significance set at *P* < 0.1 (Table 1), showed that the only presenting variable independently associated with DNS was a positive Babinski reflex (Table 2).

**Figure 1:**
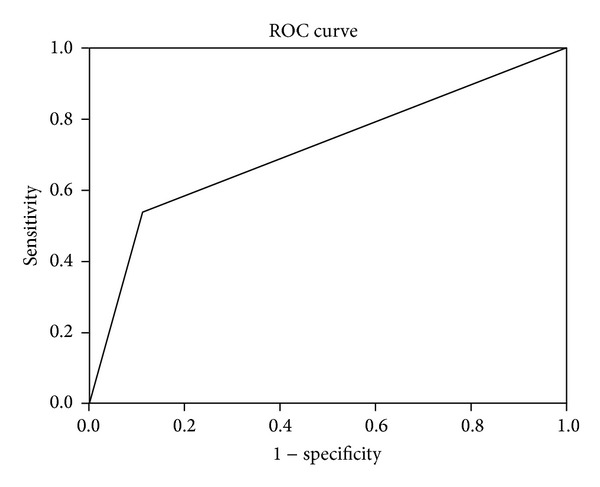
The area under the ROC curve for a positive Babinski reflex.

The presence of a positive Babinski reflex had a sensitivity of 53.8% (95% confidence interval [CI]: 26.1–79.6), specificity of 88.6% (95% CI: 83.7–92.1), positive predictive value (PPV) of 20.0% (95% CI: 9.1–37.5), and negative predictive value (NPV) of 97.3% (95% CI: 94.0–98.9) for DNS. Two hundred and seventeen patients in 223 patients without a positive Babinski sign did not have DNS; therefore, the NPV is 97.3%. In other words, only 2.7% patients (6/223) without a positive Babinski sign had DNS. The area under the ROC curve for a positive Babinski reflex was 0.712 (95% CI: 0.544–0.880), which showed good diagnostic accuracy (Figure 1).

## 4. Discussion

We found that a positive Babinski reflex was an independent predictor for DNS in Chinese patients with COP. In addition, a positive Babinski reflex was very memorable, immediately available, and applicable in clinical practice. Even when the sensitivity and PPV of a positive Babinski reflex were unsatisfactory, it had a good specificity and NPV for excluding the risk of DNS. In patients without a positive Babinski reflex, the risk for DNS was only 2.7%. This finding may help physicians make decisions about dispositions for patients with COP. In patients with a higher risk for DNS, earlier treatment and more appropriate utilization of health care services, including hyperbaric oxygen and close followup, should be considered.

There are no published reports that a Babinski reflex is associated with the prognosis of COP. Only some studies [11, 12] have proposed that a positive Babinski reflex is one of the neurologic manifestations of COP in children. A positive Babinski reflex might indicate an upper motor neuron lesion that has damaged the corticospinal tract. Occasionally, a pathological Babinski reflex is the first (and only) indication of a serious disease process, and a clearly abnormal Babinski reflex often prompts detailed neurological investigations of the brain or the spine [13].

Acute brain injury in patients who have been exposed to carbon monoxide appears to arise largely from hypoxia. Studies with mice [14], however, have shown that cerebral blood flow initially increases within minutes of carbon monoxide exposure. Blood flow remains elevated until the mouse loses consciousness, when transient cardiac compromise causes its blood pressure to decrease [14]. Because of this, autoregulation until cardiovascular homeostasis is exhausted and asphyxia or apnea begins Neurons are the central nervous system cells most vulnerable to hypoxic-ischemic insult, and they have the highest oxygen and glucose demands [14]. Acute and intense COP can lead directly to diffuse hypoxic-ischemic encephalopathy and predominantly involve the gray matter [14]. The globus pallidus is the most common site of involvement in COP. Occasionally, the caudate nucleus, putamen, and thalamus are involved in COP, but less so than the globus pallidus [14]. Involvement of the brainstem may be a reflection of more severe poisoning because its posterior structures are more resistant to hypoxia [14]. In the DNS after COP, the characteristic findings of brain MRI were small necrotic foci and demyelinating changes in the cerebral white matter and globus pallidus [4]. Demyelination with relative preservation of the axons was prominent in the frontal lobes of patients with DNS [4]. The pathophysiology of DNS may be due to direct myelinotoxic effect of carbon monoxide [8].

This investigation has several limitations. First, we did not include the level of COHb and exposure duration for analysis because these data were unavailable for all but a few patients. However, the diagnosis of COP cannot depend solely depended on the level of COHb, because COHb testing is not available in every hospital. Moreover, many patients were delayed before being sent to the hospital, which resulted in a poor correlation between the COHb and the patient's prognosis. In addition, many studies [1, 6, 9, 15] also report that the COHb level was not a predictor for neurologic sequelae. The length of time that a patient was exposed to carbon monoxide is usually unavailable and incorrect because the victim may be confused or may have lost consciousness. In addition, there are no witnesses in most circumstances of COP. Second, data were collected from a retrospective chart review. These clinical presentations or records may not have been completely documented. Third, the number of patients might have been too small to generate sufficient statistical power and the degree and characteristics of DNS were not classified in detail. Therefore, future studies with larger study populations and more detailed evaluations of DNS are warranted.

## 5. Conclusion

We conceived a retrospective study to investigate the independent predictor of DNS in Chinese patients with COP. A positive Babinski reflex was the independent predictor with a good specificity of 88.6% and NPV of 97.3% for excluding the risk of DNS. In patients without a positive Babinski reflex, the risk for DNS was only 2.7%. This finding may help physicians make decisions about dispositions for patients with COP.

## Figures and Tables

**Table 1:** Univariate analysis of variables of 258 patients of COP with delayed neuropsychiatric sequelae (DNS).

Variable	DNS^−^	DNS^+^	All	*P* value
	(*n* = 245)	(*n* = 13)	(*n* = 258)	
Age (mean ± SD)	54.4 ± 22.4	63.6 ± 22.3	54.9 ± 22.4	0.184
Age > 35 years (%)	71.2	84.6	79.5	1.000
Elderly (Age ≥ 65 years) (%)	32.7	53.8	33.7	0.136
Gender: male (%)	47.2	61.5	48.0	0.397
Systolic blood pressure (mean ± SD)	129.5 ± 22.1	136.7 ± 23.1	129.9 ± 22.1	0.293
Heart rate (mean ± SD)	87.1 ± 17.0	88.5 ± 13.7	87.2 ± 16.9	0.767
Respiratory rate (mean ± SD)	20.3 ± 2.3	21.0 ± 3.3	20.3 ± 2.4	0.287
Body temperature (mean ± SD)	36.6 ± 0.5	36.7 ± 0.9	36.6 ± 0.6	0.758
Occupational exposure (%)	12.7	23.1	13.2	0.389
Current smoker (%)	12.2	23.1	12.8	0.223
Hypertension history (%)	18.8	38.5	19.8	0.143
Diabetes history (%)	4.1	7.7	4.3	0.440
Ischemic stroke history (%)	5.7	23.1	6.6	0.045
Altered mental status (%)	33.8	61.5	35.3	0.069
Loss of consciousness (%)	61.2	84.6	62.4	0.140
Headache (%)	41.2	30.8	40.7	0.569
Dizziness (%)	57.6	30.8	56.2	0.083
Nausea or vomiting (%)	36.7	46.2	37.2	0.561
Lack of pupil reflex (%)	10.2	7.7	10.1	>0.95
Positive Babinski reflex (%)	11.4	53.8	13.6	<0.001
Incontinence (%)	23.7	38.5	24.4	0.316
Hyperbaric oxygen therapy (%)	79.3	69.2	78.7	0.483

COP: carbon monoxide poisoning; SD: standard deviation.

**Table 2:** Multivariate logistic regression modeling using univariate comparison *P* < 0.1 of 258 patients of COP with delayed neuropsychiatric sequelae.

Variable	Odds ratio (95% confidence interval)		*P* value
	Full model	Final model	
Dizziness	0.6 (0.2–2.0)	NA	
Altered mental status	1.1 (0.2–4.6)	NA	
Ischemic stroke history	2.1 (0.4–9.9)	NA	
Positive Babinski reflex	6.2 (1.7–22.7)	9.0 (2.8–28.8)	<0.001

COP: carbon monoxide poisoning; NA: not available; variable not included in the final model.
